# Optical coherence tomography findings of the peripheral retina in patients with congenital X-linked retinoschisis

**DOI:** 10.3389/fmed.2023.1280564

**Published:** 2023-11-16

**Authors:** Ayaka Nakajima, Kazuki Kuniyoshi, Chiharu Iwahashi, Fukutaro Mano, Takaaki Hayashi, Hiroyuki Kondo, Kei Mizobuchi, Itsuka Matsushita, Akiko Suga, Kazutoshi Yoshitake, Tadashi Nakano, Takeshi Iwata, Chota Matsumoto, Shunji Kusaka

**Affiliations:** ^1^Department of Ophthalmology, Kindai University Faculty of Medicine, Osaka-sayama, Japan; ^2^Department of Ophthalmology, Osaka Prefecture Saiseikai Tondabayashi Hospital, Tondabayashi, Japan; ^3^Department of Ophthalmology, The Jikei University School of Medicine, Tokyo, Japan; ^4^Department of Ophthalmology, University of Occupational and Environmental Health, Kitakyushu, Japan; ^5^Molecular and Cellular Biology Division, National Institute of Sensory Organs, NHO Tokyo Medical Center, Tokyo, Japan; ^6^Graduate School of Agricultural and Life Sciences, Faculty of Agriculture, The University of Tokyo, Tokyo, Japan

**Keywords:** congenital retinoschisis, OCT, outer nuclear layer, inner nuclear layer, inner plexiform layer, ganglion cell layer, retinal nerve fiber layer, tapetal reflex

## Abstract

**Introduction:**

Congenital X-linked retinoschisis (XLRS) presents as macular retinoschisis/degeneration in almost all patients and as peripheral retinoschisis in half the patients. Although the optical coherence tomography (OCT) findings of macular retinoschisis have been well investigated, those of peripheral retinoschisis have rarely been reported. This study aimed to report the ultra-widefield OCT findings of the peripheral retina in patients with XLRS.

**Methods:**

Medical records of 10 Japanese patients (19 eyes) with clinically and/or genetically diagnosed XLRS were retrospectively reviewed. Funduscopic, electroretinographic, and OCT findings were reviewed and evaluated. Some were also genetically evaluated for the *RS1* gene.

**Results:**

OCT of the macula revealed schises and/or cystoid changes in the inner nuclear layer (INL) and outer nuclear layer. In contrast, OCT of the peripheral retina revealed schises and/or cystoid changes in the INL in eight eyes (44%), and/or splitting in the ganglion cell layer (GCL) in 10 (56%) of the 18 eyes with clear OCT images. No schisis or cystoid changes were found in the peripheral OCT images of eight eyes (44%). A 16-year-old boy presented with retinal splitting of the GCL and INL of the inferior retina, although he had no ophthalmoscopic peripheral retinoschisis. Genetic examinations were performed on three patients, all of whom had reported missense mutations in the *RS1* gene.

**Conclusion:**

In XLRS, peripheral bullous retinoschisis results from GCL splitting in the retina. One of the 10 patients with XLRS showed intraretinal retinoschisis in the GCL in the inferior periphery, which was unremarkable on ophthalmoscopy (*occult retinoschisis*). Although both peripheral bullous retinoschisis and occult retinoschisis showed splitting/cystic changes in the GCL, further studies are needed to determine whether occult retinoschisis progresses to bullous retinoschisis.

## 1 Introduction

Congenital X-linked retinoschisis (XLRS) is one of the most common inherited retinal dystrophies in children, with a reported prevalence of 1 in 5,000–20,000 (1) and carried by 14 in 10,000 (2). Affected males present with bilaterally reduced visual acuity, often accompanied by hyperopic astigmatism (2–4). Ophthalmoscopy reveals macular retinoschisis, which is typically described as a “spoke wheel-pattern macular degeneration,” in 95%–100% and peripheral retinoschisis in 39%–50% of the affected individuals (2–4). Peripheral retinoschisis displays a thin, bullous membrane, i.e., the inner leaf of the retina, that is the superficial layer of the retina and often contains retinal vessels. Such inner leaf often ruptures, which can cause vitreous hemorrhage, and additional tears in the outer leaf/layer result in retinal detachment (2–4). A silver-gray tapetal-like reflex, i.e., a metallic sheen, can be seen in the peripheral retina of patients with XLRS (5, 6), and it seems to be related to the vitreous adhesion to the retina (5).

Macular and peripheral retinoschisis gradually progress to non-specific retinal degeneration. However, the electroretinographic (ERG) characteristics of XLRS, namely reduced b-wave in flash ERG (negative ERG), can be observed in both the early and advanced stages and aids in disease diagnosis (6).

XLRS is caused by pathogenic variants of the retinoschisin-1 (*RS1*) gene located on the short arm of chromosome X (Xp22.13). Retinoschisin is expressed in photoreceptors and bipolar cells (7) and is believed to play an important role in the adhesion between cells and materials in the retina; therefore, retinoschisin dysfunction results in congenital retinoschisis (8). To this date, *RS1* is the only identified gene causing XLRS.

Optical coherence tomography (OCT) findings of macular retinoschisis include cystoid changes and/or splitting (schisis) of the retina in the inner nuclear layer (INL), outer plexiform layer (OPL), outer nuclear layer (ONL), or retinal ganglion cell layer (GCL) in the macular region (9). However, detailed OCT findings of the peripheral retina of patients with XLRS have rarely been reported (10–13). Here, we present ultra-widefield OCT findings of the macula and peripheral retina in patients with XLRS and propose a grading system of the peripheral retinal findings.

## 2 Patients and methods

Nineteen eyes of 10 male patients clinically diagnosed with XLRS underwent OCT examination at the Kindai University Hospital (Table 1). The clinical diagnosis of XLRS was made based on the presence of the following elements: (1) fundus findings, such as macular degeneration, including spoke wheel-pattern macular degeneration and/or peripheral retinoschisis or degeneration; (2) negative flash ERG; and (3) familial history of retinoschisis, retinal detachment, or visual disturbance, suggesting an X-linked recessive trait.

**Table 1:** Clinical/genetic characteristics and OCT findings of the patients.

Family-Pt # (Figure #)	Age at exam/sex	Follow-up period (years)	Family history (trait)	*RS1* gene	Full-field flash ERG	BCVA (decimal)		Refractive errors (diopters)		Ophthalmoscopy				OCT				Others
										Macular retinoschisis		Peripheral retinoschisis		Macular retinoschisis		Peripheral retinoschisis (*Grade*; Table 2)		
						R	L	R	L	R	L	R	L	R	L	R	L	
1-1 (Figure 1)	23/M	21	Yes (XR)	NA	Negative	0.5	0.3	+2.0	−1.5	Yes	Yes	Yes (inferior)	Yes (inferior)	INL	INL	GCL, INL *Grade 1* (*superior*) *Grade 3* (*inferior*)	Image unclear	Past history of retinal detachment (L)
2-1 (Figure 2)	17/M	12	Yes (XR)	NA	Negative	0.3	0.5	+3.5	+7.0	Yes	Yes	Yes (superior)	Yes (superior)	INL	INL ONL OPL	GCL, INL *Grade 1* (*inferior*) *Grade 3* (*superior*)	GCL, INL *Grade 4* (*inferior*) *Grade 3* (*superior*)	Past history of retinal detachment (L)
3-1 (Figure 3)	16/M	4	Yes (XR)	NA	Negative	0.8	0.7	+2.5	+2.5	Yes	Yes	None	None	INL	INL	GCL, INL *Grade 1* (*superior*) *Grade 2* (*inferior*)	GCL, INL *Grade 1* (*superior*) *Grade 2* (*inferior*)	*Occult RS* in the inferior retina (R&L)
2-2	33/M	7	Yes (XR)	NA	Negative	0.5	0.07	+2.5	+2.0	Yes	Yes	None	Yes (superior)	INL ONL (GCL)	INL ONL	None (*Grade 1*)	GCL *Grade 1* (*inferior*) *Grade 3* (*superior*)	Cousin of Pt 2-1
4-1	30/M	23	Yes (XR)	c.T267A p.Tyr89X	Negative	0.2	0.3	+2.0	+2.0	Yes	Yes	Yes (inferior)	Yes (inferior)	INL	INL	GCL, INL *Grade 1* (*superior*) *Grade 3* (*inferior*)	GCL, INL *Grade 1* (*superior*) *Grade 3* (*inferior*)	Siblings
4-2 (Figure 4A)	28/M	23	Yes (XR)	c.T267A p.Tyr89X	Negative	0.3	0.2	−0.5	−0.5	Yes	Yes	None	None	INL (GCL) (OS)	INL (GCL)	None *Grade 1*	None *Grade 1*	
5-1	49/M	5	No	c.C598CT p.Arg200Cys	Negative	0.2	0.2	+2.0	+0.5	None (flecks)	None (flecks)	Yes (inferior)	None	None	None	GCL, INL *Grade 1* (*superior*) *Grade 3* (*inferior*)	None *Grade 1*	
6-1 (Figure 4B)	47/M	4	No	NA	Negative	0.4	0.3	−3.0	−3.0	None	None	None	None	INL	INL (OS) (GCL)	None *Grade 1*	None *Grade 1*	
7-1	64/M	7	Yes (XR)	NA	Negative	0.04	0.04	+6.0	+6.5	None (punctates)	None (punctates)	None (white spiculations)	None (white spiculations)	None	None	None *Grade 1*	None *Grade 1*	
8-1	51/M	23	No	NA	Negative	NLP	0.5	NA	−1.0	NA	Yes	NA	Yes (inferior)	NA	INL	NA	GCL, INL *Grade 1* (*superior*) *Grade 3* (*inferior*)	Phthisis bulbi (R)

OCT, optical coherence tomography; Pt, patient; Fig, figure; exam, examination; XR, X-linked recessive, ERG, electroretinogram; BCVA, best corrected visual acuity; R, right eye; L, left eye; M, male; NA, not available; Negative, negative flash ERG with reduced b-wave; INL, inner nuclear layer; GCL, ganglion cell layer; ONL, outer nuclear layer; OPL, outer plexiform layer; OS, outer segment of the photoreceptor; NLP, no light perception. Parentheses in OCT findings indicate slight abnormalities in the layer.

All patients underwent comprehensive ophthalmic examinations including ophthalmoscopy, fundus photography, fundus autofluorescence imaging, full-field ERG, Goldmann kinetic perimetry, and OCT. OCT was performed using Optos^®^ Silverstone SS OCT (Optos^®^ Inc., Marlborough, MA, United States) or ZEISS PLEX Elite 9000 Swept-Source OCT (Carl Zeiss Meditec AG, Jena, Germany). Scanning was performed horizontally in both the macula and periphery. A peripheral OCT scan was performed approximately 20–30° inferior/superior to the fovea. Additional vertical OCT scanning was performed in Patient 3. Finally, fundus and OCT findings of the peripheral retinas were divided into several grades (Table 2).

**Table 2:** Grading of the peripheral fundi in patients with congenital X-linked retinoschisis.

Grade	Notes	Ophthalmoscopy in the periphery	OCT in the periphery	Representative OCT images
Grade 1	No retinoschisis	Normal with/without silver-gray reflex	No schisis, no cystoid changes, and normal layer structure	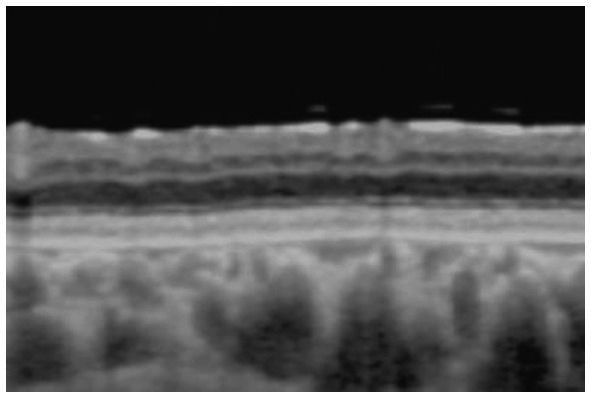
Grade 2	Occult retinoschisis	Normal with/without silver-gray reflex	Minor schisis and cystoid changes in GCL/INL	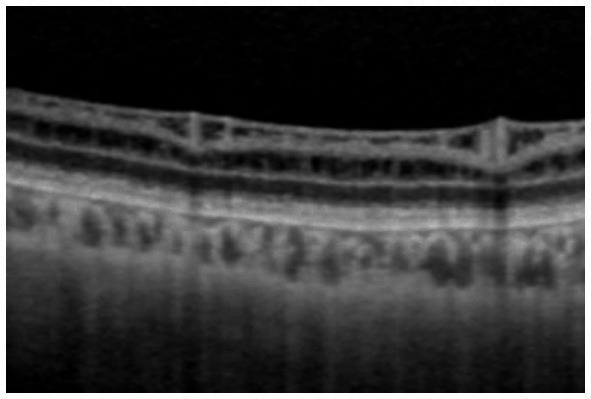
Grade 3	Retinoschisis with retinal degeneration	Retinoschisis, shallow or bullous	Splitting in GCL and cystoid changes in INL	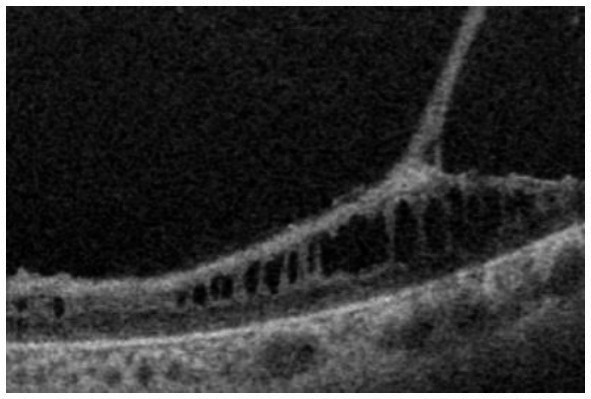
Grade 4	Retinal atrophy, most likely atrophic outer leaf of the retina	Retinal degeneration	Atrophy of the retina with no layer structure	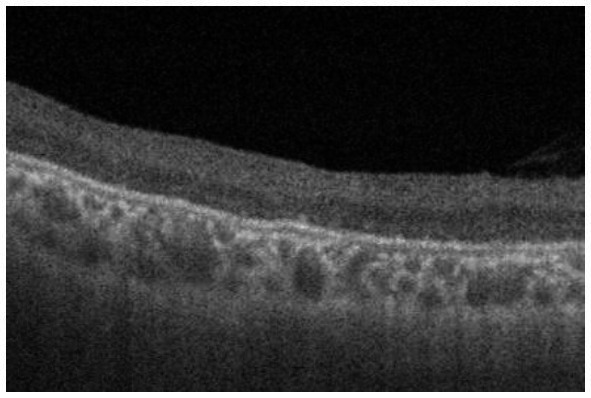
Grade +D	Retinal detachment with/without retinoschisis	Retinal detachment with/without retinoschisis	Retinal detachment with/without splitting/cystoid changes in GCL/INL	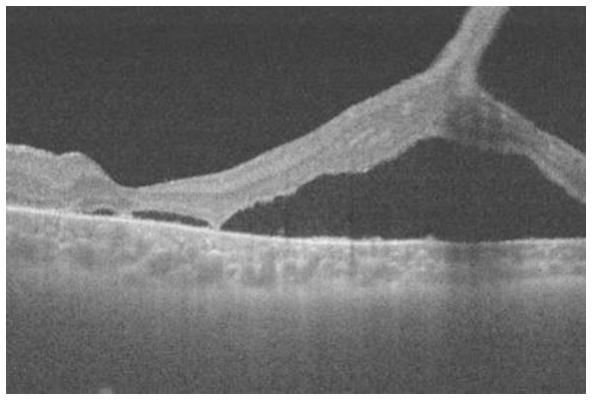

Genetic examinations were performed in three patients after obtaining written informed consent. Genomic DNA was extracted from peripheral blood and analyzed by whole exome sequencing with targeted analysis, including the *RS1* gene (at the National Institute of Sensory Organs) or Sanger sequencing targeting six coding exons of the *RS1* gene (at the Jikei University or University of Occupational and Environment Health). The RS1 transcript (NM_000330.4) served as a reference. Details of genetic examinations have been previously reported (14–16).

All procedures performed in this study involving human participants were approved by the ethics review board of each institute (Kindai University: 22-132 and R05-071, Jikei University: 24-2316997, University of Occupational and Environment Health: UOEHCRB20-148, National Institute of Sensory Organs: R22-046), and adhered to the tenets of the Declaration of Helsinki and its later amendments or comparable ethics standards.

## 3 Results

**Figure 1:**
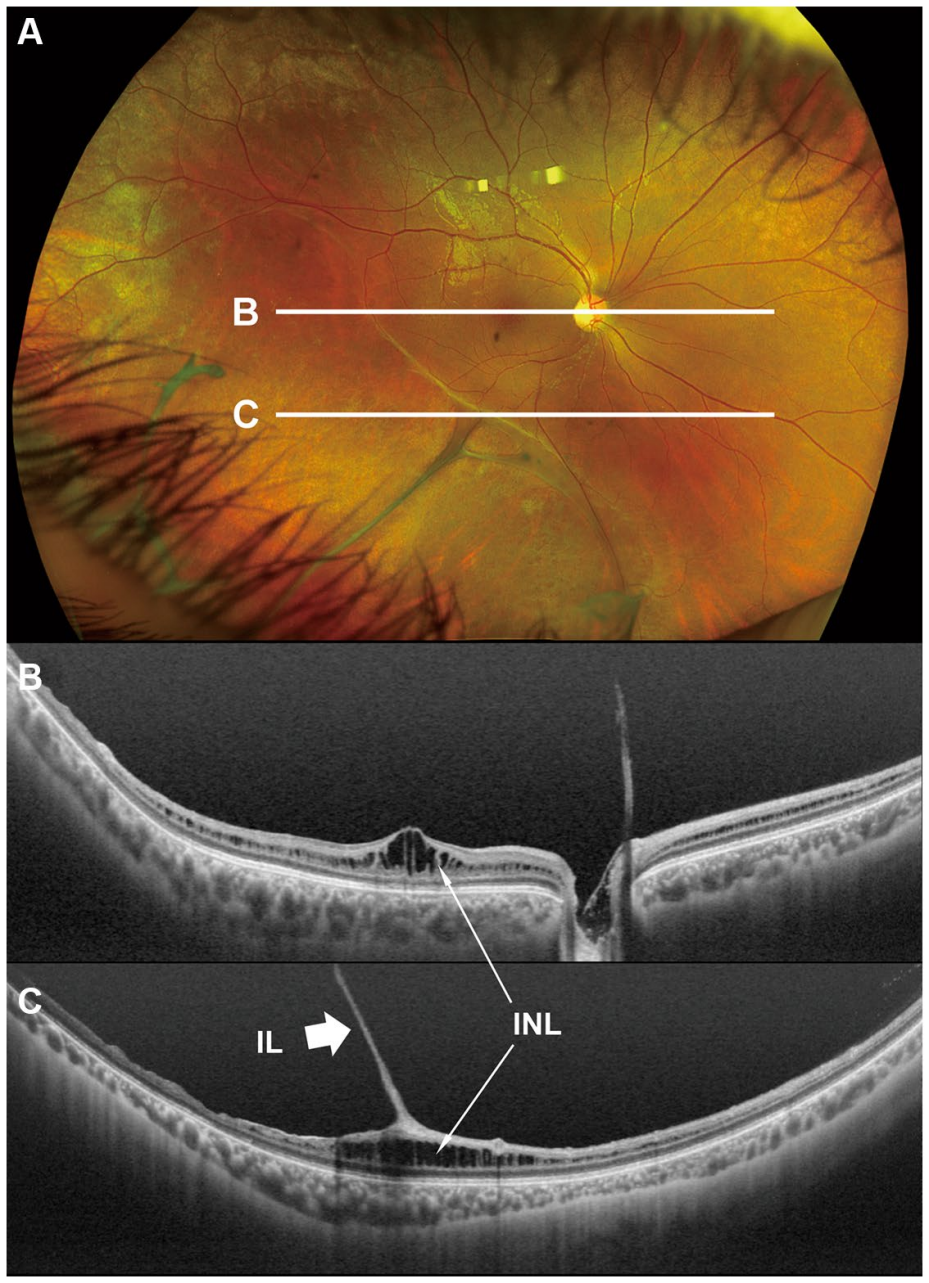
Fundus photograph **(A)** and horizontal images of optical coherence tomography (OCT) at line B **(B)** and at line C **(C)** in the fundus photograph. Bullous retinoschisis with large windows in inner leaf of the retina is seen in the inferior retina **(A)**, and the OCT image at line C shows splitting of the inner layer of the retina (**C**, *Grade 3* in Table 2). INL, inner nuclear layer; IL, inner leaf of the retina.

**Figure 2:**
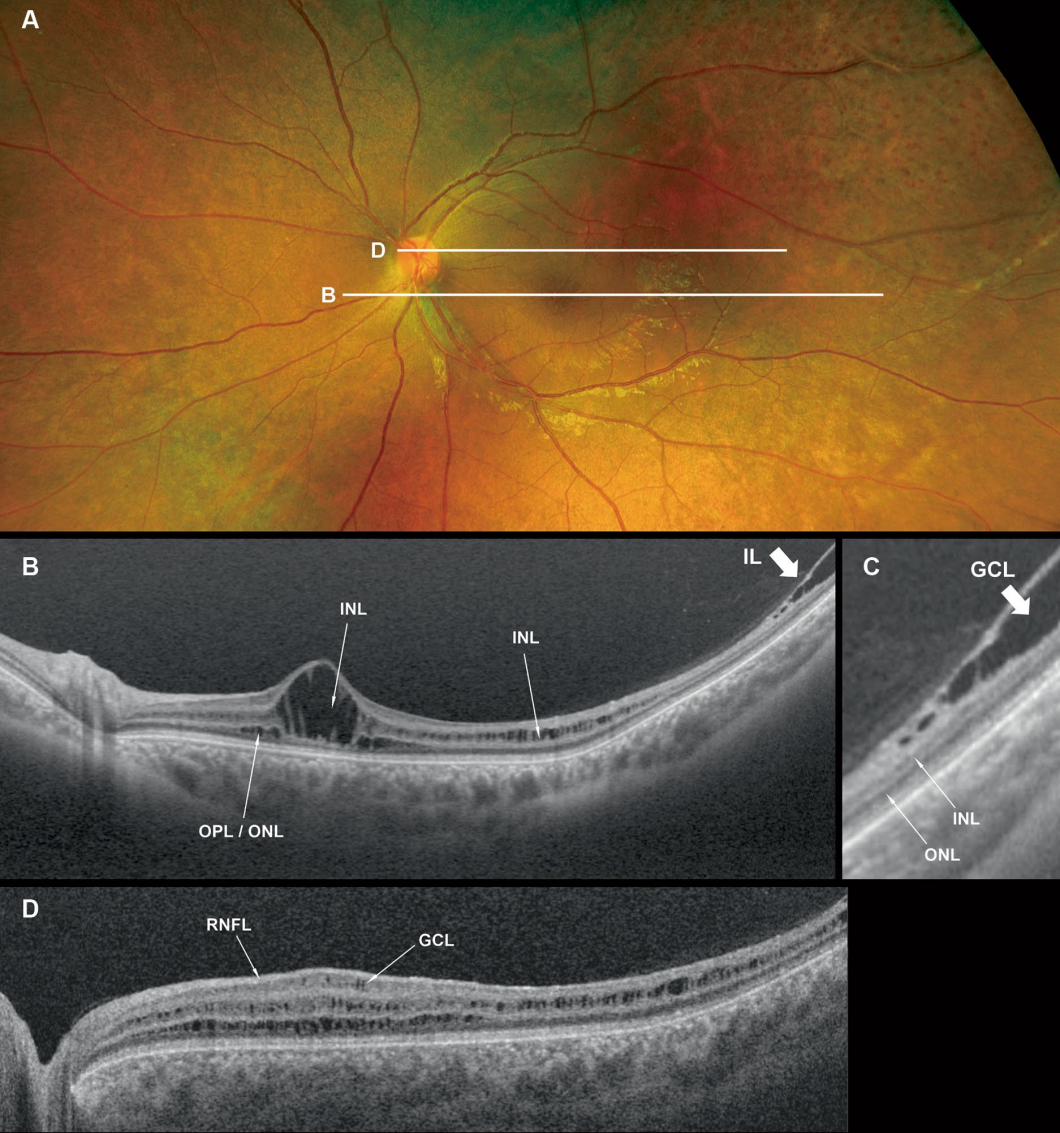
Fundus photograph **(A)**, horizontal images of OCT at line B **(B)** and line D **(D)**, and magnified image of temporal section of OCT image B **(C)**. Bullous retinoschisis is shown in the inferior retina **(A)**, and OCT image at line B shows splitting of the ganglion cell layer (**B**,**C**, *Grade 3* in Table 2). OPL, outer plexiform layer; ONL, outer nuclear layer; GCL, ganglion cell layer; RNFL, retinal nerve fiber layer.

**Figure 3:**
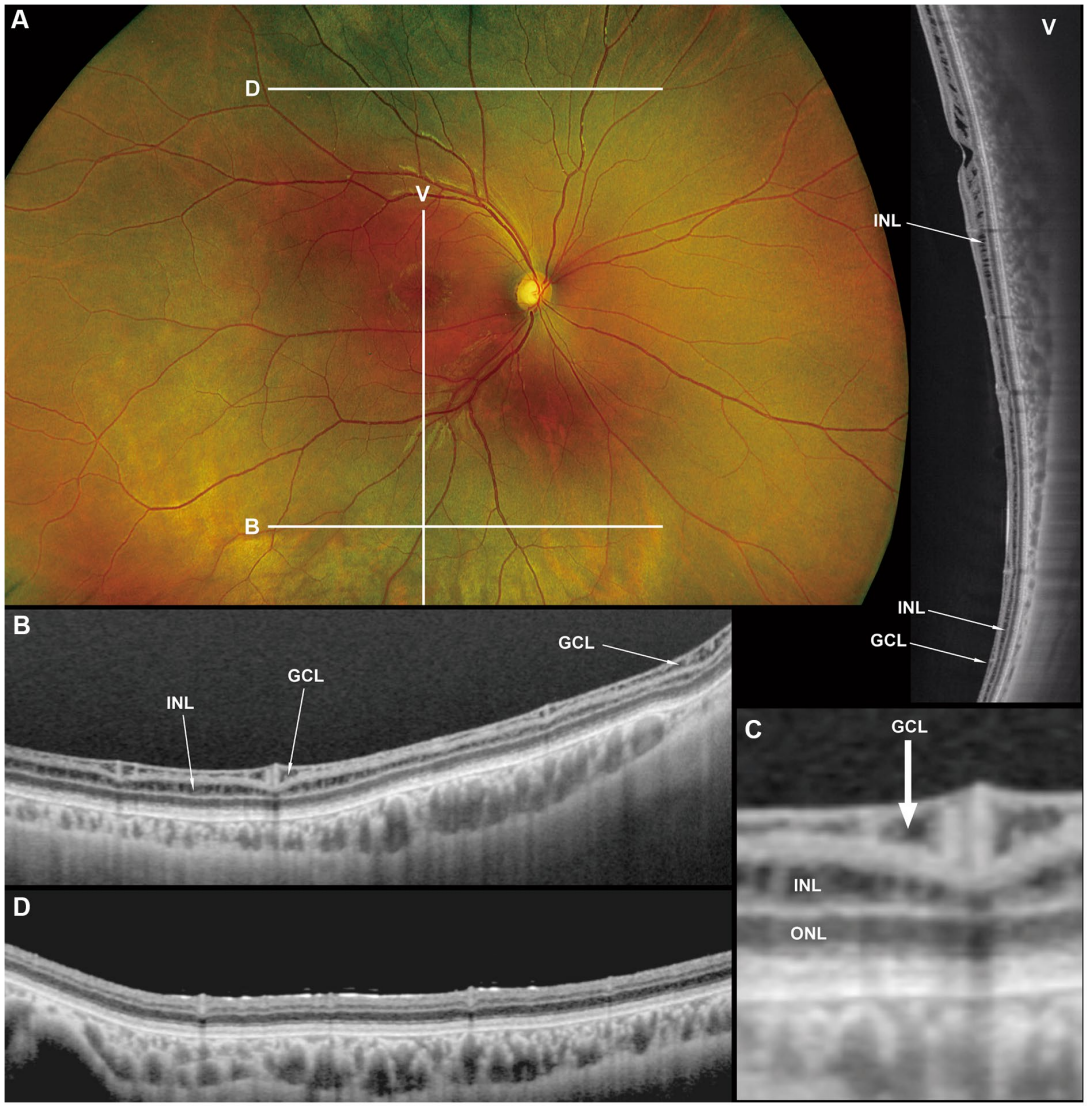
Fundus photograph **(A)**, horizontal images of OCT at line B **(B)** and line D **(D)**, magnified image of central section of OCT image B **(C)**, and vertical OCT image at line V **(V)**. OCT in the superior retina (at line D) showed no intraretinal split or cystoid changes (**D**, *Grade 1* in Table 2), however OCT in the inferior retina (at line B) shows split/cystoid changes in GCL and INL **(B,C,V)** where no retinoschisis was observed in fundus photograph **(A)**, i.e., “*occult retinoschisis*” (*Grade 2* in Table 2).

**Figure 4:**
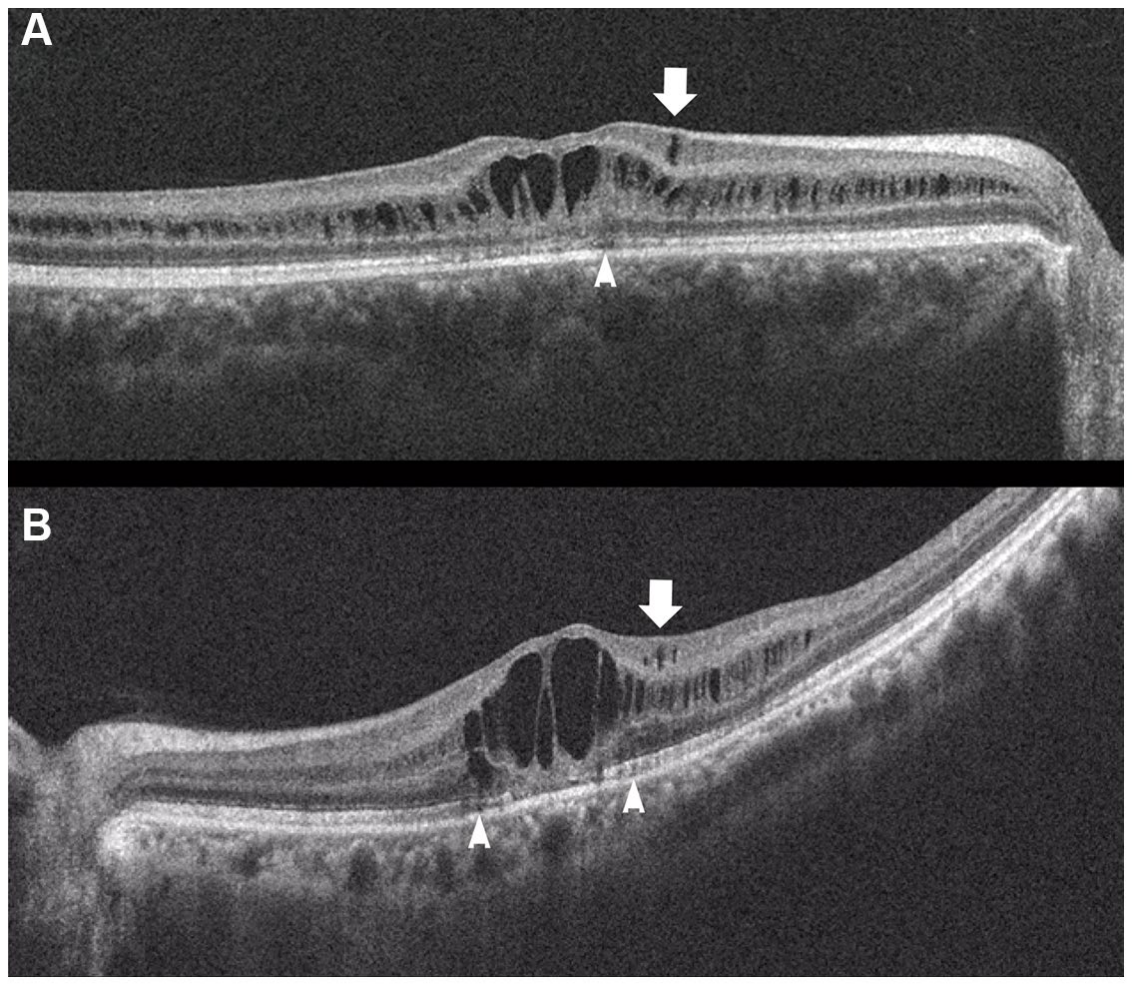
Horizontal images of OCT of the macula (**A**, Patient 4-2; and **B**, Patient 6-1 in Table 1). Cystoid changes in the GCL (arrows) are much fewer than those in the INL. Arrowheads: cystoid changes in the outer segment of the photoreceptors.

A summary of the results of the clinical and genetic examinations of the patients is shown in Table 1, and representative cases are shown in Figures 1–4 and Table 2.

At the time of examination, the age of the patients ranged from 16–64 years (median, 30 years), and they had been clinically followed up for 4–23 years. Of the 19 eyes examined, spoke wheel-pattern macular degeneration was observed in 13 (68%), punctate macular degeneration in two (11%), and flecked macula in two (11%). Peripheral retinoschisis was observed in 11 (58%) eyes. Two eyes (11%) had a history of retinal detachment and were treated with scleral buckling surgery (Patients 1-1 and 2-1 in Table 1). Both exhibited infero-temporal bullous retinoschisis with large windows in the inner leaf of the retina. Additional tear in the outer retina caused retinal detachment, although Patient 2-1 did not show any outer tear despite detailed pre- and intra-operative fundus examination.

In the macula, OCT revealed macular retinoschisis/cystoid changes in the INL in 15 eyes (79%, Figures 1B, 2B), in ONL or outer segment of the photoreceptors in four eyes (21%), and in the GCL or OPL in four eyes (21%).

In the periphery, OCT revealed that bullous retinoschisis was a result of splitting in the GCL, although many cystoid changes or intraretinal schisis were observed in the INL at the posterior edge of the retinal splitting (Figures 1C, 2B,C). In Patient 2-1, horizontal scanning superior to the fovea revealed intraretinal cystoid changes in ONL, INL, and GCL, but no changes were observed in the retinal nerve fiber layer (Figure 2D). The cystoid changes in the GCL were frequently observed in the periphery but rarely found in the macula (Figures 2C, 3B,C, and 4).

Notably, in Patient 3-1, horizontal scanning inferior to the fovea (line B in Figure 3A) revealed minor splitting of the GCL and INL (Figures 3B,C), although ophthalmoscopy showed no peripheral retinoschisis (Figure 3A). However, horizontal OCT scanning superior to the fovea (line D in Figure 3A) showed no cystoid changes or retinal splitting (Figure 3D). An additional vertical OCT scan revealed cystoid changes/minor splitting in the INL continuously throughout the macula to the inferior retina. However, minor splitting of the GCL was observed in the peripheral retina but not in the posterior retina (Figure 3V).

Our OCT findings of the peripheral retina in the patients with XLRS were divided into several grades (Table 2), as follows: *Grade 1*, no retinoschisis in both ophthalmoscopy and OCT; *Grade 2*, no retinoschisis in ophthalmoscopy but intraretinal split/cystoid changes in OCT (“*occult retinoschisis*”); *Grade 3*, peripheral retinoschisis with/without retinal degeneration; *Grade 4*, atrophic retina with no layered structure on OCT images, that is the most likely an atrophic outer leaf of the retina. And *Grade + D*, retinal detachment with any grade of XLRS, e.g., *Grade 3 + D* represents retinal detachment in patients with Grade 3 XLRS. The representative OCT images are shown in Table 2.

Genetic examinations revealed previously reported missense mutations in *RS1* gene in Patients 4-1, 4-2 and 5-1 (Table 1) (17).

## 4 Discussion

This study reported detailed ultra wide-field OCT findings in the peripheral retina of patients with XLRS. OCT revealed that split and/or cystoid changes in the INL were common to both the macula and peripheral retina. However, cystoid changes in the GCL were observed in the peripheral retina, but rarely in the macula (Figures 2–4). Peripheral retinoschisis resulted from splitting of the GCL (Figures 1C, 2C).

Dysfunction of *RS1* (retinoschisin 1) causes a structurally vulnerable retina and results in congenital retinoschisis (6). Retinoschisin is expressed in photoreceptors and bipolar cells; however, it is not expressed in the GCL (5), which cannot explain the splitting of the GCL in the peripheral retina. Further studies are required to investigate the precise mechanisms underlying peripheral retinoschisis.

Interestingly, ultra wide-field OCT revealed intraretinal retinoschisis in the inferior retina of Patient 3-1, where no retinoschisis was found by detailed fundus examination using an indirect ophthalmoscope or slit-lamp microscopy with a magnified fundus lens. Therefore, we propose the term “*occult retinoschisis*” to describe the optical coherent tomographic retinoschisis in Patient 3 (Figure 3B and Table 2).

Similar findings were reported by Gregori et al. (10) in 2013. They demonstrated minor splitting in the INL and GCL in the superior/inferior retina around the vascular arcades in a 13-year-old boy who presented shallow inferotemporal retinoschisis in the fundus. In our study, we successfully recorded OCT images outside of the vascular arcade using ultra wide-field OCT. Similar findings were also reported in the macular retinoschisis, in which lamellar retinoschisis was reported in the OCT images of a boy with XLRS who showed a clinically normal macula (18).

More than half a century ago, Yanoff et al. (19, 20) reported intraretinal retinoschisis in the inferior retina in an enucleated eye with XLRS, without bullous retinoschisis. They described the intraretinal retinoschisis as “early splitting within GCL or RNFL (19, 20).” Authors believe that the “occult retinoschisis” is the living image of the Yanoff-described “early splitting within GCL or RNFL.”

Both the Yanoff-described “early splitting within GCL or RNFL” and occult retinoschisis were found in the inferior retina (Figures 3B,V), while the peripheral retinoschisis was also frequently seen in the inferior retina in patients with XLRS (Figures 1A, 2A). These findings suggest that early splitting within the GCL or RNFL, or occult retinoschisis may be related to the development of peripheral retinoschisis. Therefore, patients with XLRS and occult retinoschisis (*Grade 2*) may have a higher risk of developing peripheral retinoschisis (*Grade 3*) than those without peripheral occult retinoschisis (*Grade 1*). However, whether occult retinoschisis can progress to peripheral retinoschisis, spontaneously resolve, or both, remains unclear, as only Patient 3 showed occult retinoschisis, and no ultra wide-field OCT-follow up was presented in this study.

The silver-gray tapetal reflex, a characteristic of the peripheral retina in patients with XLRS (5, 6), seems to have no relationship with occult retinoschisis, as such reflex observed in the periphery, both with and without occult retinoschisis (*Grades 1* and *2*, Figures 3A,B,D).

The limitations of this study include a small cohort size and the absence of wide-field OCT-follow up in the patients. Further longitudinal studies are required in a larger cohort with XLRS.

Finally, we propose a grading system for the peripheral retina in patients with XLRS (Table 2). We believe that this grading is useful for clinical research on patients with XLRS. Further studies on the natural course of XLRS are needed to investigate alterations among grades in the peripheral retina.

## 5 Conclusion

Ultra wide-field OCT of the periphery revealed GCL splitting in patients with XLRS. Splitting of the GCL was observed not only in peripheral retinoschisis but also in the ophthalmoscopically normal retina (*occult retinoschisis*). Grading of the peripheral retina using ultra wide-field OCT may be useful for evaluating disease progression in patients with XLRS.

## Data availability statement

The datasets presented in this study can be found in online repositories. The names of the repository/repositories and accession number(s) can be found in the article/supplementary material.

## Ethics statement

The studies involving humans were approved by Ethics Review Board of Kindai University, The Jikei University, University of Occupational and Environment Health, and National Institute of Sensory Organs. The studies were conducted in accordance with the local legislation and institutional requirements. Written informed consent for participation in this study was provided by the participants' legal guardians/next of kin.

## Author contributions

AN: Writing – original draft, Conceptualization, Formal analysis. KK: Writing – original draft, Conceptualization, Data curation, Formal analysis, Funding acquisition, Investigation, Methodology, Project administration, Resources, Supervision, Validation, Visualization, Writing – review & editing. CI: Conceptualization, Formal analysis, Investigation, Methodology, Project administration, Supervision, Validation, Writing – review & editing. FM: Conceptualization, Data curation, Formal analysis, Investigation, Methodology, Project administration, Resources, Software, Supervision, Validation, Visualization, Writing – review & editing. TH: Writing – original draft, Conceptualization, Data curation, Formal analysis, Funding acquisition, Investigation, Methodology, Project administration, Resources, Supervision, Validation, Writing – review & editing. HK: Conceptualization, Data curation, Formal analysis, Funding acquisition, Investigation, Methodology, Project administration, Resources, Supervision, Validation, Writing – review & editing. KM: Validation, Writing – review & editing, Conceptualization, Data curation, Formal analysis, Investigation, Methodology, Project administration, Supervision. IM: Conceptualization, Data curation, Formal analysis, Investigation, Methodology, Project administration, Resources, Supervision, Validation, Writing – review & editing. AS: Conceptualization, Data curation, Formal analysis, Investigation, Methodology, Project administration, Resources, Supervision, Validation, Writing – review & editing. KY: Conceptualization, Data curation, Formal analysis, Investigation, Methodology, Project administration, Resources, Supervision, Validation, Writing – review & editing. TN: Conceptualization, Formal analysis, Funding acquisition, Investigation, Methodology, Project administration, Resources, Supervision, Validation, Writing – review & editing. TI: Conceptualization, Data curation, Formal analysis, Funding acquisition, Investigation, Methodology, Project administration, Resources, Software, Supervision, Validation, Writing – review & editing. CM: Conceptualization, Formal analysis, Investigation, Methodology, Project administration, Supervision, Writing – review & editing. SK: Conceptualization, Formal analysis, Investigation, Methodology, Project administration, Supervision, Validation, Writing – review & editing.
